# Effects of Quintuply-Fortified Salt on the Micronutrient Status of Children 12 to 59 Months of Age in Punjab, India: Results from a Randomized, Community-Based, Household Trial

**DOI:** 10.1016/j.tjnut.2026.101573

**Published:** 2026-05-05

**Authors:** Julie M Long, Yvonne E Goh, Mona Duggal, Reena Das, Mari S Manger, Manu Jamwal, Bidhi L Singh, Gurjinder Kaur Brar, Jamie Westcott, Lauren Thompson, Charles D Arnold, Nancy F Krebs, Kenneth H Brown, Christine M McDonald

**Affiliations:** 1 Department of Pediatrics, Section of Nutrition, University of Colorado School of Medicine, Aurora, CO, United States; 2 Department of Pediatrics, Division of Gastroenterology, Hepatology and Nutrition, University of California, San Francisco, CA, United States; 3 Post Graduate Institute of Medical Education & Research, Chandigarh, India; 4 Department of Nutrition and Institute for Global Nutrition, University of California, Davis, CA, United States

**Keywords:** micronutrients, fortification, child nutrition, salt, India

## Abstract

**Background:**

Micronutrient deficiencies remain prevalent among preschool-aged children (PSC) in India. Quintuply-fortified salt (QFS) is one of many potential interventions to improve micronutrient intake and status at the population level.

**Objectives:**

To determine the effect of QFS compared with iodized salt (IS) for 12 mo on the micronutrient status of PSC.

**Methods:**

This was a substudy of a double-blinded, household-randomized, controlled, community-based trial involving nonpregnant females of reproductive age whose households were randomly assigned to receive: *1*) QFS with zinc, vitamin B-12, folic acid, iodine, and iron as encapsulated ferrous fumarate (eFF-QFS); *2*) QFS with the same micronutrients, but iron as encapsulated ferric pyrophosphate plus ethylenediaminetetraacetic acid (eFePP-QFS); or *3*) IS. The micronutrient status of 470 PSC (aged 12–59 mo) residing in these households was assessed at enrollment, 6 mo, and 12 mo. Continuous outcomes were analyzed with linear regression and reported as means or geometric mean ratios, and binary outcomes were analyzed with logistic regression and reported as odds ratios.

**Results:**

At baseline, the prevalence of anemia, iron deficiency anemia, hypozincemia, vitamin B-12 insufficiency, and folate deficiency was 35%, 30%, 14%, 17%, and 5.5%, respectively. Effects of QFS at 6 and 12 mo were greatest for vitamin B-12 and folate. At 12 mo, the eFePP-QFS group had higher serum vitamin B-12 [geometric mean ratio (GMR) = 1.16, 95% confidence interval (CI) = 1.03, 1.30], serum folate (GMR = 1.29, 95% CI = 1.09, 1.53), and red blood cell folate (GMR = 1.21, 95% CI = 1.05, 1.39) concentrations compared with the IS group. Effects were similar among the 2 QFS groups. There were no significant differences in serum zinc, ferritin, hemoglobin, or urinary iodine between groups at 6 and 12 mo.

**Conclusions:**

Preschool children consuming QFS for 12 mo demonstrated greater improvements in vitamin B-12 and folate status compared with children consuming IS. QFS may be a useful vehicle to address micronutrient deficiencies, especially vitamin B-12 and folate, in this population.

## Introduction

The global prevalence of undernutrition among children <5 y has decreased in the past 20 y, but in India, high rates persist [1]. According to India’s Comprehensive National Nutrition Survey (CNNS; 2016–2018), 35% of children are stunted, and 17% are wasted [2]. The CNNS also found multiple micronutrient (MN) deficiencies among young children: 41% are anemic, and the national prevalences of iron deficiency, zinc deficiency, vitamin B-12 deficiency, and folate deficiency are 32%, 19%, 14%, and 23%, respectively [2]. Young children are particularly vulnerable to many MN deficiencies, given the increased nutrient requirements to support rapid growth and development during the early years of life [[3], [4], [5], [6]]. Thus, deficiencies in these MNs are associated with several adverse child health outcomes, including impaired linear growth, suboptimal neurobehavioral development, and increased incidence of diarrhea and other infectious morbidity.

Public health interventions, such as micronutrient fortification of staple foods and condiments, can effectively improve micronutrient status at the population level [7,8]. In India, an estimated 93% of households already use adequately iodized salt (IS) [9]. In addition, India has recently scaled up the distribution of doubly-fortified salt (DFS) containing iodine and iron through social protection programs in some states [10]. Extensive research conducted in various settings has shown that DFS significantly improves iron status in nutritionally vulnerable groups [[11], [12], [13]]. New technology has made it possible to fortify salt with additional micronutrients [14]. Results of our recently-completed randomized controlled trial that assessed 2 types of quintuply-fortified salt (QFS) with iron, zinc, vitamin B-12, folate, and iodine compared with IS among women of reproductive age (WRA) in Punjab, India showed that consumption of QFS resulted in sizable improvements in vitamin B-12 and folate status [15]. Modest, but inconsistent, improvements in iron and zinc status were also observed.

An advantage of using a fortification vehicle, such as salt, which is consumed by all household members, is that everyone in the household may benefit nutritionally. The objective of this substudy was to evaluate the effects of QFS containing iron, zinc, folic acid, vitamin B-12, and iodine on the micronutrient status of preschool-aged children (PSC) whose households participated in the parent trial in Punjab, India. Children consumed the study salt for 12 mo, and biomarkers of micronutrient status were measured at baseline, 6 mo, and 12 mo.

## Methods

### Study design

**FIGURE 1:**
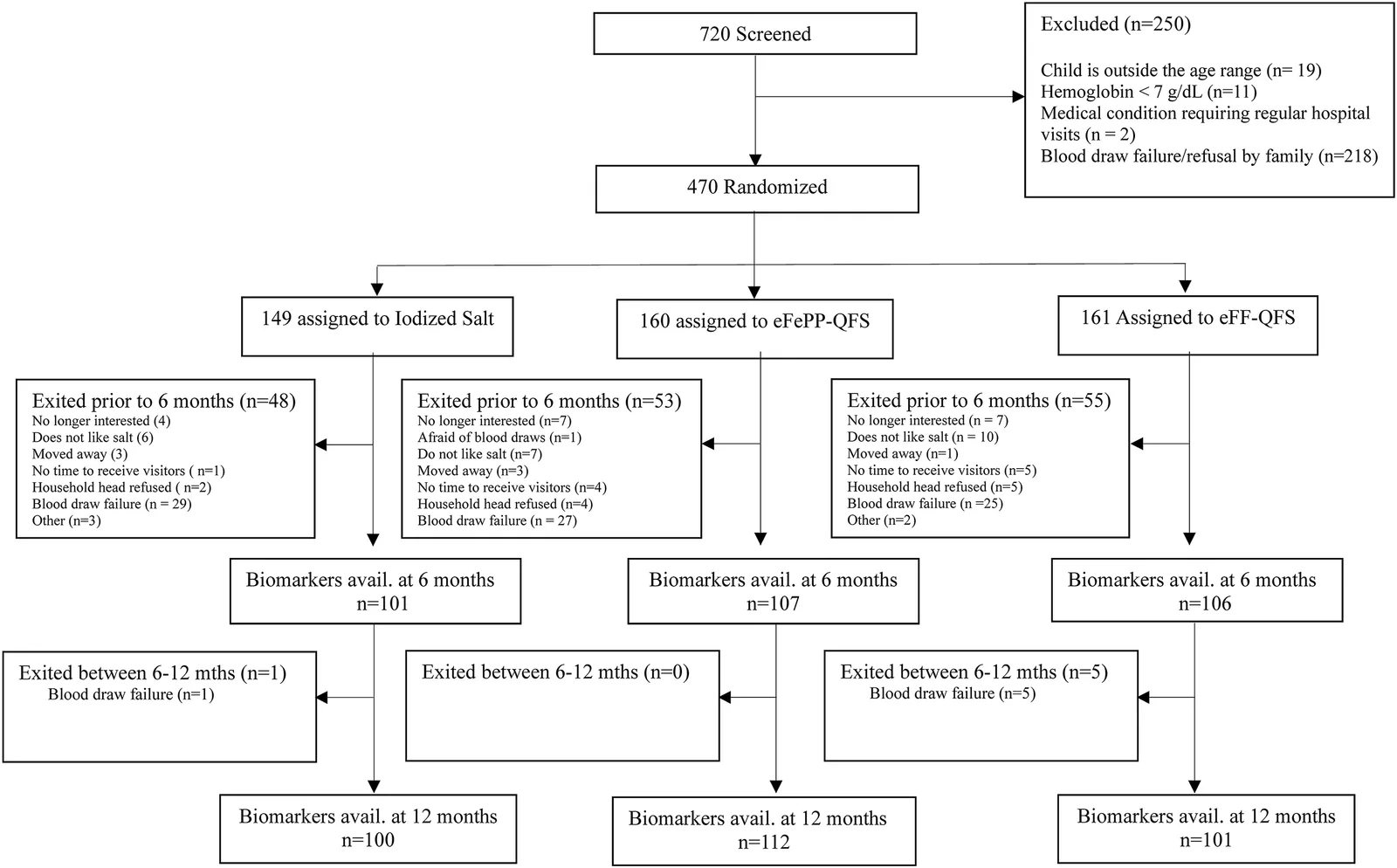
Flowchart of trial participants. eFePP-QFS, quintuply-fortified salt with iron as encapsulated ferric pyrophosphate plus EDTA, zinc, vitamin B-12, folic acid, and iodine; eFF-QFS, quintuply-fortified salt with iron as encapsulated ferrous fumarate, zinc, vitamin B-12, folic acid, and iodine.

This substudy was nested within the parent double-blinded, randomized, controlled, community-based trial of QFS with iron in the form of encapsulated ferric pyrophosphate plus EDTA (eFePP-QFS) compared with QFS with iron in the form of encapsulated ferrous fumarate (eFF-QFS) compared with IS among nonpregnant WRA in the Mohali district of Punjab, India [15,16]. We hypothesized that children enrolled in the eFePP-QFS and eFF-QFS groups would have greater improvements in biomarkers of iron, zinc, vitamin B-12, and folate compared with the IS group after 6 and 12 mo of consuming the study salts. This substudy was conducted from October 2022 through March 2023. Four hundred and seventy children whose households were participating in the parent study were assigned to the same salt intervention arm and followed for 12 mo (Figure 1).

### Participants

Children were eligible and enrolled in the study if they met the following inclusion criteria: *1*) aged 12–59 mo at the time of enrollment, *2*) mother or primary female caregiver was enrolled into the parent trial, *3*) family was a permanent resident of the study village with no plans to move or travel outside the village for >4 wk over the next 12 mo, and *4*) the child’s mother or primary female caregiver was willing to use the salt provided by the study as the primary source of the household’s discretionary salt for the course of the study. Exclusion criteria included: *1*) severe anemia (defined as a hemoglobin concentration <7 g/dL) and *2*) a serious medical problem that interfered with the child’s eating practices.

### Randomization and masking

Children were screened for eligibility at the same time as their mothers. If the mother–child pair met the screening criteria, then consent was obtained and the enrollment questionnaire was completed. Mother–child pairs were then scheduled for biochemical assessments and anthropometric measurements. If the blood draw was successful, mother–child pairs were randomly assigned to one of the following intervention groups: eFePP-QFS, eFF-QFS, or IS. Although very uncommon, if 2 children from the same household were eligible for the study, only 1 child was enrolled. The decision was made with a random coin toss. The study statistician generated the group assignment list in a ratio of 1:1:1 and included the randomization block number, envelope number, and color-coded group assignment. Block randomization of 12 ensured even distribution of the groups throughout the study enrollment, and participants were assigned consecutive unassigned identification numbers. Mothers selected from 12 sealed opaque envelopes that contained color-coded group assignments. Once the card was selected, the mother–child pair was assigned its own identification number. This process was repeated until all 12 envelopes from the block were selected.

To ensure participants and researchers remained blinded throughout the study, 2 colors were assigned to each study salt for a total of 6 color-coded salts. The randomization and color assignment were completed by a researcher not involved in the study. The salts were packaged in opaque bags to ensure that researchers and participants could not tell the differences in the physical appearance of the salt. Even after the bags were opened, no physical differences in the study salts could be observed.

### Study salts and adherence

The micronutrient content of the eFF-QFS was as follows: iron in the form of eFF (1.34 mg iron/g salt), zinc in the form of zinc oxide (1.4 mg zinc/g salt), vitamin B-12 (0.6 μg vitamin B-12/g salt), and folic acid (52.4 μg folic acid/g salt). eFePP-QFS had the same micronutrient content, except that iron was in the form of ferric pyrophosphate, and EDTA was included as an enhancer of absorption. All 3 salts were iodized with 30 μg of iodine per gram of salt.

JVS Foods/Wella Nutralogicals produced the micronutrient premix and packaged the study salts in 500 g color-coded durable bags. Each household received a monthly allotment of salt depending on household size: households with 1 to 5 members received two 500 g packages; those with 6 to 11 members received three 500 g packages; and those with ≥12 members received four 500 g packages. If participant households ran out of salt before their next monthly visit, an additional 500 g was provided. Mothers were instructed to use study salt for all recipes and meals. In addition, participants were asked not to share or sell the study salt with anyone outside the family home or purchase any other salt. Education was also provided to participants about what to expect with QFS salt use and that minimal changes to food color were acceptable and safe.

Adherence to the assigned study salt was monitored by assessing salt disappearance at the household level during monthly home visits conducted by the research team. To calculate the amount of salt used in the previous month, the amount of leftover salt was weighed and subtracted from the amount of salt provided at the previous monthly visit. To estimate the amount of salt consumed by the study child, we applied the adult male equivalent method to calculate the child-equivalent level of consumption per day [17].

### Anthropometry and collection of clinical specimens

At the baseline, 6 mo, and 12 mo visits, children arrived with their mothers at a central location in the village between 6:30 and 10:30. Researchers requested that children avoid eating or drinking anything for a minimum of 8 h leading up to the blood draw. For children who were still breastfeeding, mothers were not given specific instructions about withholding breastmilk before the blood draw. Anthropometric measurements were performed by 2 trained research assistants according to WHO protocols [18,19].

A trained phlebotomist collected 7 mL of whole blood from every child using a trace-element-free Safety-Multifly and 2 S-Monovettes containing EDTA or no additional additives. All procedures were completed to avoid zinc contamination [20]. All monovettes were placed into an electronic portable cooler, maintained at 4°C, and transported to the field laboratory for processing. In addition, a 50 mL morning spot urine sample was collected from each child and transported to the field laboratory for processing.

### Laboratory analyses

One-hundred microliters of whole blood were aliquoted from the EDTA tube into each of 2 cryovials containing 1% ascorbic acid solution for the subsequent analysis of red blood cell (RBC) folate. The remaining blood was centrifuged at 2000 × *g* for 10 min to separate plasma for homocysteine analysis. After being at room temperature for 30 min, blood samples in serum collection tubes were centrifuged at 2000 × *g* for 10 min. Serum zinc concentration was analyzed using atomic absorption spectroscopy. Serum ferritin, vitamin B-12, and thyroglobulin concentrations were measured by electrochemiluminescence immunoassays on a Cobas E411 Analyzer (Roche Diagnostics), and soluble transferrin receptor (sTfR), α1-acid glycoprotein, and C-reactive protein, and urinary creatinine were measured by particle-enhanced immunoturbidimetric assay on a Cobas 8000 Analyzer (Roche Diagnostics). Urinary iodine was measured using human urinary iodine ELISA kits (Shanghai Coon Koon Biotech). RBC and serum folate were analyzed using the US Centers for Disease Control and Prevention (CDC) microbiologic assay reagents with chloramphenicol-resistant strains of *Lactobacillus rhamnosus* culture and a 5-methyltetrahydrofolate calibrator, and the laboratory participated in the CDC performance verification [21]. Plasma homocysteine was analyzed using the fully automated ADVIA Centaur XP immunoassay system (Siemens Healthcare Solutions). Serum holo-transcobalamin (holo-TC) was measured using ELISA (Axis-Shield Diagnostics), and methylmalonic acid (MMA) was analyzed via gas chromatography mass spectrometry (Agilent Technologies). All relevant quality control data for the laboratory analyses were previously published as Supplementary Material in the study’s primary results paper [15].

### Binary outcomes

The following cutoffs were used to assess the prevalence of micronutrient deficiency and insufficiency. Anemia was defined as a hemoglobin concentration <10.5 g/dL for children aged 6 to 23 mo and <11 g/dL for children aged 24 to 59 mo [22]. Iron deficiency was defined as an inflammation-adjusted ferritin concentration of <12 μg/dL, and iron deficiency anemia was defined as the presence of anemia and either inflammation-adjusted ferritin <12 μg/dL or inflammation-adjusted sTfR >8.3 mg/L [3,[22], [23], [24]]. Children were identified as having hypozincemia if fasting serum zinc was <65 μg/dL [4]. Vitamin B-12 insufficiency was defined as a serum vitamin B-12 <165 pmol/L (children aged 12–23 mo) or <183 pmol/L (children aged 24–59 mo) [6]. Participants were identified as having low holo-TC if serum holo-TC was <19 pmol/L (children aged 12–23 mo) or <29 pmol/L (children aged 24–59 mo), high homocysteine if serum homocysteine was >15 μmol/L, or high MMA if serum MMA was >0.83 μmol/L (children aged 12–23 mo) or >0.30 μmol/L (children aged 24–59 mo) [6]. Folate deficiency was assessed by serum folate and RBC folate. Deficiency was defined as serum folate <10 nmol/L and RBC folate <305 nmol/L [5,25]. Iodine deficiency was defined as a median urinary iodine concentration <100 μg/L [26].

### Sample size and statistical power

The sample size for the parent study was based on an effect size difference of 0.3 SD for all continuous micronutrient biomarker outcomes across the 3 study arms, and 238 females were required per arm [15]. One hundred and fifty-six PSC were enrolled into each arm of the trial, with a goal of obtaining an effective sample size of ≥130 PSC per arm. This sample size enabled the detection of a change in the prevalence of anemia from 40% to 23%, of iron deficiency from 67% to 49%, of zinc deficiency from 21% to 8%, of vitamin B-12 insufficiency from 17% to 5% and of folate deficiency from 10% to 1%, assuming an *α* of 0.05, power of 80%, and an attrition rate of 20% [2]. These sample sizes translated into a detectable effect size of 0.35 SD units for the change in the mean concentrations of the primary micronutrient biomarkers from enrollment to the end of the 12 mo intervention period among the PSC subgroup.

### Statistical analyses

Baseline demographic and biochemical data were presented as mean and SD or median and interquartile ranges for normally and nonnormally-distributed data, respectively. In assessing intervention effects, inverse probability of censoring weighting (IPCW) was applied in all models due to higher-than-anticipated attrition rates [27]. Continuous outcome biomarkers were assessed using linear regression, adjusting for appropriate baseline measures, and binary biomarker outcomes were assessed using logistic regression, also adjusting for baseline measures. Skewed outcomes were log-transformed for analysis. Pairwise comparisons were conducted between the 3 intervention arms with a Hommel correction to maintain the family-wise error rate [28]. We also assessed effect modification by baseline status and salt intake tertile by including an interaction term between intervention arm and the effect modifier in the IPCW models. Salt disappearance was calculated from household salt disappearance data collected at the monthly visits. The child equivalent was converted based on the number of occupants in the household and standard assumptions of weight and total energy expenditure [17,29]. Statistical analyses were completed using STATA (version 16.1, StataCorp) or R (v4.4, R Core Team).

### Ethical approval

Written informed consent was provided by the mother or caregiver of every participant before commencing study procedures. This study was reviewed and approved by the institutional review boards at the University of California, San Francisco, Postgraduate Institute of Medical Education and Research, Chandigarh, and the Colorado Multiple Institutional Review Board. This study was registered with clinicaltrials.gov, NCT05166980.

## Results

This substudy screened 720 children, and it enrolled and randomly assigned 470 children aged 12 to 59 mo (Figure 1) to one of the three study salts. One hundred and sixty children were assigned to the eFePP-QFS group, 161 assigned to the eFF-QFS group, and 149 children assigned to the IS group. Of those 470 enrolled, 314 children completed the 6 mo study visit, and 313 children completed the 12 mo study visit. Attrition rates did not statistically vary among intervention groups, with 70% of children in the eFePP-QFS group completing the 12 mo visit compared with 63% of children in the eFF-QFS group and 67% of children in the IS group. Reasons for dropout included unsuccessful initial blood draws, being no longer interested in study participation, dislike of the study salt, moving away from the study catchment, household head refused participation, and lack of time to receive visitors (Figure 1).

Slightly less than half (46.4%) of the enrolled child participants were females, and the mean ± SD age at enrollment was 39.4 ± 12.2 mo (Table 1). Approximately one-third (34.6%) of children were currently breastfeeding at enrollment. The prevalences of stunting and wasting were 14.3% and 6.8%, respectively. Mean ± SD maternal age was 29.9 ± 4.5 y, and BMI was 25.2 ± 4.8 kg/m^2^. Most females had a middle or secondary school education or greater. The mean ± SD number of people in a household was 6.4 ± 2.3, and 96.6% of children resided in food-secure households. At baseline, the prevalence among children of anemia was 35.3%, iron deficiency was 30.0%, iron deficiency anemia was 30.3%, hypozincemia was 14.1%, vitamin B-12 insufficiency was 17.0%, and folate deficiency (based on RBC folate) was 3.6%. We examined per capita household salt disappearance data and estimated that discretionary salt intake per child equivalent was ∼3.5 g/d and did not significantly vary across groups.

**TABLE 1:** Background characteristics and micronutrient status of study participants at enrollment by intervention group^1^.

	Iodized salt (*n* = 149)	eFePP-QFS (*n* = 160)	eFF-QFS (*n* = 161)
Participant characteristics			
Sex, female	61 (40.9)	74 (46.3)	83 (51.6)
Age, mo	38.4 ± 12.2	40.2 ± 12.0	39.6 ± 12.3
Currently breastfeeding	45 (36.6)	41 (32.5)	47 (34.6)
Weight-for-age *z*-score	–0.72 ± 1.14	–0.70 ± 1.12	–0.68 ± 1.06
Underweight^2^	15 (10.1)	16 (10.0)	14 (8.7)
Length-for-age *z*-score	–0.83 ± 1.47	–0.78 ± 1.6	–0.83 ± 1.24
Stunting^2^	24 (16.1)	22 (13.8)	21 (13.0)
Weight-for-length *z*-score	–0.35 ± 1.12	–0.39 ± 1.23	–0.32 ± 1.17
Wasting^2^	7 (4.7)	12 (7.5)	13 (8.1)
BMI-for-age *z*-score	–0.28 ± 1.17	–0.33 ± 1.24	–0.25 ± 1.21
Maternal characteristics			
Age, y	29.5 ± 4.4	30.0 ± 4.4	30.2 ± 4.6
BMI, kg/m^2^	25.5 ± 4.6	25.0 ± 4.8	25.2 ± 5.1
Maternal education			
No formal education	0	3 (1.9)	2 (1.3)
Primary school	16 (11.0)	17 (10.8)	12 (7.8)
Middle and/or secondary	101 (69.7)	106 (67.5)	110 (71.4)
Diploma or higher	28 (19.3)	31 (19.7)	30 (18.5)
Mother currently works for income	13 (8.7)	12 (7.5)	7 (4.4)
Household characteristics^3^			
Household size	6.5 ± 2.3	6.6 ± 2.5	6.1 ± 2.1
Household is headed by a female	27 (18.1)	27 (16.9)	29 (18.0)
Household assets index^4^	1.9 ± 0.9	1.8 ± 0.9	1.8 ± 0.8
Household is food secure^5^	143 (96.0)	155 (98.9)	156 (96.9)
Household has electricity	149 (100)	160 (100)	160 (99.4)
Household has piped water	149 (100)	160 (100)	160 (99.4)
Household uses gas for cooking	148 (99.3)	158 (98.8)	159 (98.8)
Household has a car	55 (36.9)	59 (36.9)	52 (32.3)
Participants’ micronutrient status			
Hemoglobin, g/dL	11.2 ± 1.3	11.3 ± 1.4	11.4 ± 1.4
Hemoglobin <10.5 g/dL or <11 g/dL^6^	59 (39.6)	55 (34.4)	52 (32.3)
Iron deficiency anemia^6^	50 (33.6)	46 (29.1)	45 (28.3)
Iron			
Ferritin, μg/L^7^	7.1 (4.0, 14.0)	8.9 (4.4, 18.0)	9.3 (4.9, 18.7)
Ferritin <12 μg/L^7^	100 (67.1)	101 (63.1)	100 (62.1)
sTfR, mg/L^7^	5.6 (4.2, 7.6)	5.6 (4.3, 8.3)	5.6 (4.3, 7.4)
sTfR >8.3 mg/L^7^	36 (24.2)	38 (24.1)	35 (22.0)
Body iron stores, mg/kg^8^	–0.6 ± 4.3	–0.2 ± 4.6	0.0 ± 4.3
Zinc			
Serum zinc, μg/dL	75.9 ± 11.4	76.9 ± 12.2	76.0 ± 10.4
Serum zinc <65 μg/dL	7 (10.0)	13 (16.0)	12 (15.8)
Vitamin B-12			
Serum B-12, pmol/L	370 (244, 564)	349 (225, 547)	362 (247, 522)
Serum B-12 <165 or <183 pmol/L	25 (16.8)	27 (16.9)	28 (17.4)
Holo-transcobalamin, pmol/L	58.1 (43.0, 89.0)	55.6 (38.8, 92.8)	53.2 (31.3, 79.3)
Holo-transcobalamin <19 or <29 pmol/L	11 (12.9)	13 (13.7)	15 (16.9)
Homocysteine, μmol/L	16.0 (12.2, 20.6)	15.3 (12.1, 19.3)	15.5 (12.4, 18.8)
Homocysteine >15 μmol/L	84 (56.4)	82 (51.2)	86 (53.4)
Methylmalonic acid, μmol/L	0.7 (0.5, 1.1)	0.8 (0.5, 1.4)	0.7 (0.4, 1.3)
Methylmalonic acid >0.83 or >0.30 μmol/L	76 (89.4)	86 (90.5)	76 (85.4)
Folate			
Serum folate, nmol/L	15.3 (10.8, 19.9)	16.2 (12.0, 22.8)	16.1 (11.4, 24.1)
Serum folate <7 nmol/L	6 (4.0)	2 (1.2)	9 (5.6)
Red blood cell folate, nmol/L	529 (410, 632)	529 (438, 686)	498 (396, 639)
Red blood cell folate <305 nmol/L	7 (4.7)	7 (4.4)	12 (7.5)
Iodine			
Thyroglobulin, μg/L	38.0 (26.2, 54.8)	42.2 (30.0, 58.9)	42.0 (29.8, 56.9)
Urinary iodine, μg/L	561 (336, 720)	539 (323, 681)	514 (334, 686)
Urinary iodine <100 μg/L	0	0	1 (0.6)
Inflammation			
C-reactive protein, mg/L	0.4 (0.2, 1.7)	0.4 (0.2, 1.6)	0.3 (0.2, 1.3)
C-reactive protein >5 mg/L	16 (10.7)	17 (10.8)	17 (10.7)
α1-Acid glycoprotein, g/L	0.8 (0.6, 1.1)	0.7 (0.6, 1.0)	0.8 (0.6, 1.1)
α1-Acid glycoprotein >1 g/L	46 (30.9)	38 (24.1)	49 (30.8)

Abbreviations: BRINDA, Biomarkers Reflecting Inflammation and Nutritional Determinants of Anemia; eFePP-QFS, quintuply-fortified salt with iron as encapsulated ferric pyrophosphate plus EDTA, zinc, vitamin B-12, folic acid, and iodine; eFF-QFS, quintuply-fortified salt with iron as encapsulated ferrous fumarate, zinc, vitamin B-12, folic acid, and iodine; sTfR, soluble transferrin receptor.

1 Values are mean ± SD, *n* (%), or median (Q1, Q3) for nonnormally-distributed micronutrient biomarkers.

2 Underweight, stunting, and wasting are based on <–2.00 SDs [18].

3 A household was defined as a group of people who are primarily dependent on an individual person, currently eat from the same pot, and live under the same roof or in the same compound.

4 Calculated using binary responses (yes or no) to ownership of 12 household assets. The first component of the principal component analysis was used to compute household index means per group.

5 Calculated using the Household Food Insecurity Access Scale [37].

6 Anemia: Hb <10.5 g/dL for ages 6–23 mo and <11 g/dL for ages 24–59 mo; iron deficiency anemia: inflammation-adjusted ferritin <12 μg/L or sTfR >8.3 mg/L and anemia [3,22].

7 Ferritin and sTfR values were adjusted for inflammation using the BRINDA regression equations [22,23].

8 Body iron stores were calculated using the equation: −[log(sTfR/ferritin) − 2.8229]/0.1207. The values indicate the iron surplus in stores (positive values) or the iron deficit in tissues (negative values) [3].

At 6 mo, both the eFePP-QFS and eFF-QFS groups exhibited improvements in all biomarkers of vitamin B-12, including serum vitamin B-12, holo-transcobalamin, homocysteine, and MMA compared with the IS group (Table 2). These improvements were sustained at 12 mo: the geometric mean of serum vitamin B-12 was 16% higher for the eFePP-QFS group compared with the IS group, and 22% higher for the eFF-QFS group compared with the IS group. However, vitamin B-12 insufficiency was too rare to assess as a binary outcome at 6 and 12 mo (Table 3).

**TABLE 2:** Effect of quintuply-fortified salt on hemoglobin and micronutrient status of preschool-aged children in Punjab, India^1^.

	Biomarker concentrations			Mean differences or geometric mean ratios (95% CI)^2^		
	Iodized salt	eFePP-QFS	eFF-QFS	eFePP-QFS compared with iodized salt	eFF-QFS compared with iodized salt	eFF-QFS compared with eFePP-QFS
*n* at 6 mo	69–101	84–107	80–106	—	—	—
*n* at 12 mo	73–100	76–112	71–101	—	—	—
Hemoglobin, g/dL						
6 mo	11.3 ± 1.3	11.6 ± 1.3	11.6 ± 1.3	0.02 (–0.27, 0.31)	–0.09 (–0.39, 0.20)	–0.11 (–0.39, 0.17)
12 mo	11.6 ± 1.3	11.8 ± 1.2	11.9 ± 1.2	0.22 (–0.04, 0.49)	0.12 (–0.16, 0.40)	–0.11 (–0.37, 0.16)
Iron						
Ferritin, μg/L^3^						
6 mo	14.1 (8.6, 23.3)	14.6 (9.2, 26.2)	18.6 (9.7, 27.6)	0.95 (0.77, 1.17)	0.96 (0.80, 1.15)	1.00 (0.82, 1.23)
12 mo	11.9 (6.5, 18.2)	12.4 (6.9, 20.8)	18.0 (8.4, 26.3)	0.94 (0.76, 1.17)	1.06 (0.85, 1.33)	1.13 (0.91, 1.39)
sTfR, mg/L^3^						
6 mo	5.0 (3.9, 7.0)	5.0 (3.9, 6.7)	4.6 (3.9, 6.2)	1.04 (0.93, 1.17)	0.97 (0.88, 1.07)	0.93 (0.83, 1.04)
12 mo	5.1 (4.3, 7.5)	4.9 (4.0, 6.2)	4.8 (3.9, 5.9)	0.89 (0.80, 1.01)	0.87 (0.77, 0.98)∗	0.98 (0.87, 1.09)
Body iron stores, mg/kg 4						
6 mo	1.9 ± 3.5	2.3 ± 3.5	2.7 ± 3.6	–0.38 (–1.23, 0.46)	–0.08 (–0.89, 0.73)	0.31 (–0.61, 1.22)
12 mo	0.9 ± 3.6	1.7 ± 3.5	2.6 ± 3.9	0.11 (–0.87, 1.10)	0.62 (–0.42, 1.65)	0.50 (–0.51, 1.51)
Zinc						
Serum zinc, μg/dL						
6 mo	77.2 ± 10.9	77.7 ± 11.2	78.4 ± 9.9	3.90 (–4.52, 12.33)	6.74 (–1.51, 14.99)	2.84 (–5.52, 11.19)
12 mo	79.0 ± 11.6	76.9 ± 12.5	77.5 ± 12.8	3.47 (–3.40, 10.35)	0.43 (–6.46, 11.32)	–3.04 (–9.76, 3.68)
Vitamin B-12						
Serum B-12, pmol/L						
6 mo	371.9 (267.2, 505.0)	421.3 (306.7, 571.5)	438.9 (316.7, 619.5)	1.13 (0.99, 1.30)	1.23 (1.07, 1.40)∗∗	1.08 (0.96, 1.22)
12 mo	374.0 (258.9, 529.2)	462.4 (336.9, 614.9)	451.9 (326.8, 627.8)	1.16 (1.03, 1.30)∗∗	1.22 (1.08, 1.37)∗∗	1.05 (0.94, 1.17)
Holo-transcobalamin, pmol/L						
6 mo	51.0 (38.2, 87.7)	59.3 (35.2, 86.7)	61.6 (41.7, 94.5)	0.98 (0.83, 1.15)	1.15 (1.00, 1.32)∗	1.18 (1.02, 1.36)∗
12 mo	50.8 (34.6, 66.6)	55.2 (38.2, 78.9)	65.8 (44.8, 91.6)	1.07 (0.93, 1.24)	1.29 (1.11, 1.49)∗∗	1.20 (1.05, 1.37)∗∗
Homocysteine, μmol/L						
6 mo	17.0 (13.9, 20.2)	14.7 (12.7, 18.5)	14.7 (12.4, 18.1)	0.91 (0.84, 0.98)∗∗	0.88 (0.82, 0.95)∗∗	0.97 (0.90, 1.04)
12 mo	13.5 (11.5, 15.8)	12.1 (10.2, 14.6)	11.9 (9.6, 14.4)	0.91 (0.82, 1.00)	0.87 (0.80, 0.96)∗∗	0.96 (0.89, 1.04)
Methylmalonic acid, μmol/L						
6 mo	0.8 (0.6, 1.2)	0.7 (0.6, 1.0)	0.8 (0.6, 1.1)	0.90 (0.80, 1.01)	0.93 (0.83, 1.05)	1.04 (0.94, 1.15)
12 mo	0.9 (0.5, 1.2)	0.7 (0.5, 0.8)	0.5 (0.4, 0.8)	0.82 (0.71, 0.94)∗∗	0.78 (0.68, 0.91)∗∗	0.96 (0.84, 1.09)
Folate						
Serum folate, nmol/L						
6 mo	12.8 (9.4, 17.8)	20.2 (13.0, 29.4)	19.6 (14.0, 29.5)	1.34 (1.15, 1.55)∗∗∗	1.31 (1.14, 1.51)∗∗∗	0.98 (0.86, 1.12)
12 mo	11.7 (8.6, 18.1)	16.7 (12.4, 26.1)	18.1 (12.6, 27.4)	1.29 (1.09, 1.53)∗∗	1.36 (1.13, 1.63)∗∗	1.05 (0.88, 1.25)
Red blood cell folate, nmol/L						
6 mo	466.7 (328.9, 667.1)	647.8 (463.3, 951.8)	620.9 (455.7, 825.7)	1.38 (1.18, 1.62)∗∗∗	1.29 (1.09, 1.53)∗∗	0.94 (0.79, 1.11)
12 mo	470.0 (383.5, 619.4)	568.1 (452.1, 790.1)	608.1 (436.8, 814.2)	1.21 (1.05, 1.39)∗∗	1.23 (1.06, 1.43)∗∗	1.02 (0.90, 1.15)
Iodine						
Thyroglobulin, μg/L						
6 mo	29.3 (20.2, 44.1)	33.8 (23.5, 46.5)	32.0 (23.4, 44.3)	0.94 (0.85, 1.04)	0.98 (0.88, 1.09)	1.05 (0.95, 1.16)
12 mo	26.9 (19.4, 38.2)	31.8 (23.0, 43.9)	32.3 (22.3, 41.2)	0.98 (0.88, 1.10)	1.04 (0.92, 1.17)	1.06 (0.94, 1.18)
Urinary iodine, μg/L						
6 mo	464.3 (369.5, 644.8)	444.2 (355.9, 581.4)	450.7 (298.9, 673.7)	0.93 (0.81, 1.06)	0.94 (0.81, 1.08)	1.01 (0.88, 1.16)
12 mo	615.1 (425.5, 911.4)	681.9 (426.0, 949.2)	678.8 (423.1, 937.8)	1.02 (0.88, 1.19)	1.04 (0.89, 1.22)	1.02 (0.89, 1.17)

Abbreviations: BRINDA, Biomarkers Reflecting Inflammation and Nutritional Determinants of Anemia; CI, confidence interval; eFePP-QFS, quintuply-fortified salt with iron as encapsulated ferric pyrophosphate plus EDTA, zinc, vitamin B-12, folic acid, and iodine; eFF-QFS, quintuply-fortified salt with iron as encapsulated ferrous fumarate, zinc, vitamin B-12, folic acid, and iodine; sTfR, soluble transferrin receptor.

1 Values are mean ± SD for normally-distributed variables, median (Q1, Q3) for nonnormally-distributed variables which were log-transformed for analysis.

2 Comparisons between intervention groups are mean differences and 95% CI for hemoglobin (g/dL), body iron stores (mg/kg), and zinc (μg/dL) concentrations, which were normally distributed. Geometric mean ratios and 95% CI were reported for all other biomarkers. All analyses adjusted for the baseline measure of the outcome. ∗ *P* < 0.10, ∗∗ *P* < 0.05, ∗∗∗ *P* < 0.001. *P* values were adjusted for multiplicity via the Hommel method [28].

3 Ferritin and sTfR values were adjusted for inflammation using the BRINDA regression equations [22,23].

4 Body iron stores were calculated using the equation –[log(sTfR/ferritin) − 2.8229]/0.1207. The values indicate the iron surplus in stores (positive values) or the iron deficit in tissues (negative values) [3].

**TABLE 3:** Effect of quintuply-fortified salt on anemia and micronutrient deficiencies among preschool-aged children in Punjab, India.

	Prevalence, *n* (%)			Odds ratios (95% CI)^1^		
	Iodized salt	eFePP-QFS	eFF-QFS	eFePP-QFS compared with iodized salt	eFF-QFS compared with iodized salt	eFF-QFS compared with FePP-QFS
n at 6 mo	69–101	84–107	80–106	—	—	—
n at 12 mo	73–100	76–112	71–101	—	—	—
Anemia^2^						
6 mo	31 (30.7)	34 (31.8)	30 (28.3)	2.12 (0.91, 4.90)	1.84 (0.77, 4.39)	0.87 (0.36, 2.08)
12 mo	22 (22.0)	23 (20.5)	20 (19.8)	0.95 (0.30, 2.96)	2.78 (0.68, 11.32)	2.94 (0.66, 13.04)
Iron						
Ferritin <12 μg/L^3^						
6 mo	44 (43.6)	45 (42.1)	36 (34.0)	1.12 (0.50, 2.50)	0.82 (0.36, 1.87)	0.73 (0.32, 1.68)
12 mo	52 (52.0)	55 (49.1)	36 (35.6)	1.09 (0.48, 2.48)	0.69 (0.30, 1.61)	0.64 (0.28, 1.43)
sTfR >8.3 mg/L^3^						
6 mo	19 (18.8)	19 (17.8)	14 (13.2)	3.53 (0.90, 13.89)	0.64 (0.18, 2.23)	0.18 (0.03, 0.95)
12 mo	21 (21.0)	11 (9.8)	12 (11.9)	0.14 (0.03, 0.64)∗∗	0.26 (0.06, 1.14)	1.95 (0.47, 8.02)
Iron deficiency anemia^4^						
6 mo	23 (22.8)	27 (25.2)	20 (18.9)	3.68 (1.18, 11.51)∗	2.01 (0.66, 6.11)	0.55 (0.19, 1.61)
12 mo	20 (20.0)	15 (13.4)	14 (13.9)	0.47 (0.13, 1.64)	1.02 (0.22, 4.65)	2.18 (0.50, 9.45)
Zinc						
Serum zinc <65 μg/dL						
6 mo	7 (10.1)	8 (9.5)	7 (8.8)	0.75 (0.01, 40.72)	5.85 (0.08, 417.39)	7.80 (0.02, 3643)
12 mo	9 (12.3)	14 (18.4)	9 (12.7)	3.35 (0.35, 32.12)	0.59 (0.12, 2.80)	0.18 (0.02, 2.02)
Vitamin B-12						
Serum B-12^5^						
6 mo	9 (8.9)	4 (3.7)	3 (2.8)	Too rare	Too rare	Too rare
12 mo	6 (6.0)	4 (3.6)	0 (0.0)	Too rare	Too rare	Too rare
Holo-transcobalamin^6^						
6 mo	11 (12.9)	13 (13.7)	5 (5.6)	0.23 (0.03, 2.13)	0.03 (0.00, 0.68)∗	0.13 (0.02, 0.94)∗
12 mo	13 (15.3)	11 (11.6)	9 (10.1)	0.38 (0.06, 2.25)	0.26 (0.02, 3.69)	0.68 (0.10, 4.39)
Homocysteine >15 μmol/L						
6 mo	69 (68.3)	49 (45.8)	49 (46.2)	0.20 (0.09, 0.44)∗∗∗	0.20 (0.09, 0.44)∗∗∗	1.03 (0.51, 2.07)
12 mo	31 (31.0)	24 (21.4)	21 (20.8)	0.27 (0.11, 0.65)∗∗	0.24 (0.10, 0.56)∗∗	0.89 (0.37, 2.14)
Methylmalonic acid^7^						
6 mo	81 (95.3)	92 (96.8)	88 (98.9)	Too rare	Too rare	Too rare
12 mo	81 (95.3)	93 (97.9)	76 (85.4)	Too rare	Too rare	Too rare
Folate						
Serum folate <7 nmol/L						
6 mo	5 (5.0)	2 (1.9)	1 (0.9)	Too rare	Too rare	Too rare
12 mo	14 (14.0)	6 (5.6)	3 (3.0)	0.07 (0.01, 0.50)∗∗	0.05 (0.00, 2.86)	0.72 (0.03, 19.06)
RBC folate <305 nmol/L						
6 mo	20 (19.8)	5 (4.7)	7 (6.6)	0.03 (0.00, 0.27)∗∗	0.08 (0.01, 0.66)∗∗	3.20 (0.40, 25.98)
12 mo	8 (8.0)	6 (5.4)	4 (4.0)	0.09 (0.00, 3.76)	0.03 (0.00, 9.38)	0.36 (0.01, 18.98)

Abbreviations: BRINDA, Biomarkers Reflecting Inflammation and Nutritional Determinants of Anemia; CI, confidence interval; eFePP-QFS, quintuply-fortified salt with iron as encapsulated ferric pyrophosphate plus EDTA, zinc, vitamin B-12, folic acid, and iodine; eFF-QFS, quintuply-fortified salt with iron as encapsulated ferrous fumarate, zinc, vitamin B-12, folic acid, and iodine; RBC, red blood cell; sTfR, soluble transferrin receptor.

1 All analyses adjusted for the baseline measure of the outcome. ∗ *P* < 0.10, ∗∗ *P* < 0.05, ∗∗∗ *P* < 0.001. *P* values were adjusted for multiplicity via the Hommel method [71].

2 6–23 mo: hemoglobin <10.5 g/dL and 24–59 mo: hemoglobin <11 g/dL.

3 Ferritin and sTfR values were adjusted for inflammation using the BRINDA regression equations [23,24].

4 Iron deficiency anemia was defined as inflammation-adjusted ferritin <15 μg/L or inflammation-adjusted sTfR >8.3 mg/L and Hb <12 g/dL.

5 12–23 mo: serum vitamin B-12 <165 pmol/L and 24–59 mo: serum vitamin B-12 <183 pmol/L.

6 12–23 mo: holo-transcobalamin <19 pmol/L and 24–59 mo: holo-transcobalamin <29 pmol/L.

7 12–23 mo: methylmalonic acid >0.83 μmol/L and 24–59 mo: methylmalonic acid >0.30 μmol/L.

Results for biomarkers of folate mirrored those of vitamin B-12. Both the eFePP-QFS and eFF-QFS groups had improvements in serum folate and RBC folate at 6 and 12 mo compared with the IS group. At 12 mo, the mean serum folate ranged from 29% to 36% higher, and the mean RBC folate ranged from 21% to 23% higher in the 2 QFS groups compared with IS, and the QFS groups did not differ from each other. We observed that participants consuming the eFePP-QFS and eFF-QFS salts had lower prevalences of folate deficiency; however, estimation of precise odds ratios was not feasible for all comparisons because of the small number of events in the QFS groups.

There were no differences in mean hemoglobin or the geometric mean of inflammation-adjusted serum ferritin concentrations at 6 or 12 mo or adjusted sTfR at 6 mo. However, the geometric mean of sTfR was 11% to 13% lower in both QFS groups compared with the IS group at 12 mo. There were no differences in the odds of anemia or iron deficiency anemia by intervention group at 6 or 12 mo (Table 3). There were no group-wise differences in serum ferritin, serum zinc, urinary iodine, or serum thyroglobulin at 6 or 12 mo. Likewise, there were no differences in the odds of hypozincemia or iodine deficiency at either time point.

For effect modification by baseline micronutrient status, we observed differential results for serum homocysteine at 6 mo and serum MMA at 12 mo with stronger intervention effects among those who had high values for these biomarkers at enrollment (Supplementary Figure 1). Qualitative evaluations suggested stronger vitamin B-12 intervention effects among those who had elevated concentrations of homocysteine and MMA at baseline. In addition, when evaluating effect modification by salt intake tertile, we found only 2 of 34 tests had *P* < 0.1 (less than expected by chance), and further investigation showed inconsistent patterning.

## Discussion

This double-blinded randomized, controlled, community-based study of PSC in Punjab, India, demonstrated that consumption of QFS for 12 mo improved vitamin B-12 and folate status for children who consumed the eFePP-QFS and eFF-QFS compared with children who consumed IS. These findings are encouraging because they suggest that salt fortification could be an effective strategy for improving the vitamin B-12 and folate status of young children, which can have important implications for overall child health and development.

We did not observe any differences in urinary iodine or thyroglobulin concentrations across the 3 groups, which indicates that the incorporation of additional micronutrients did not interfere with the benefits that standard IS provides. This study also assessed the efficacy of 2 forms of iron delivered in salt: eFePP + EDTA and eFF. It was hypothesized that eFePP + EDTA would be a superior fortificant compared with eFF due to the white color, and inclusion of EDTA to enhance the bioavailability of iron [30]. However, the current study showed no consistent impact of either QFS formulation on children’s iron status. Adherence to study salt did not differ by study arm, and effect modification by adherence did not reveal any differential impact by intake. Although there was limited effect modification by baseline micronutrient status, the effects observed were greater among participants with high values of homocysteine and MMA at enrollment, which indicates that children with poorer vitamin B-12 status at baseline had the greatest benefit.

To our knowledge, this is the first randomized clinical trial (RCT) to assess the effectiveness of 2 types of QFS compared with an active control of IS in PSC. The findings from this child substudy are similar to those of the parent trial involving nonpregnant WRA. The most pronounced effect on micronutrient status was observed for vitamin B-12 and folate. It is hypothesized that the improvements in vitamin B-12 and folate are related to the dose of fortification for these 2 micronutrients relative to the estimated average requirement (EAR) values. Daily discretionary intake of study salt provided PSC was ∼2.1 μg/d (210% of the Indian EAR for vitamin B-12), 182 μg/d [164–187% (age dependent) of the EAR for dietary folate], and 4.9 mg/d [95–175% (age dependent) of the EAR for dietary zinc]. By contrast, the level of iron fortification provided just 4.6 mg/d (58–77% of the EAR). In the parent study, females who consumed either eFePP-QFS or eFF-QFS had improved vitamin B12 and folate status at 6 and 12 mo compared with females who consumed IS [15]. This is similar to what we observed in this study—an increase in the geometric mean of serum vitamin B-12 and RBC folate, suggesting a positive impact of consuming fortified salt. In addition, at 6 mo, females in the eFF-QFS group had a reduced odds of hypozincemia compared with the IS group in the parent study. In this current study, we did not observe any differences in serum zinc among PSC at 6 or 12 mo.

Previous randomized controlled studies assessing the impact of DFS with iodine and iron in children reported improvements in biomarkers of iron status, including serum ferritin, sTfR, body iron stores, and hemoglobin after 6 to 9 mo of consumption [[11], [12], [13]]. In this current study, we did not observe any differences in hemoglobin or ferritin and total iron body stores between groups. This may be due to the relatively lower levels of iron fortification used: our study provided an average of ∼1.3 mg of iron per gram of salt, whereas previous published studies typically provided 2 to 3 mg of iron per gram of salt [11,13,15]. In addition, the diets of this study population are high in phytate, which may have inhibited iron absorption [[31], [32], [33]]. It is also possible that children were experiencing subclinical inflammation due to environmental enteric dysfunction or poor sanitation and hygiene practices practices that were stimulating hepcidin production and preventing iron absorption [34].

Several studies have assessed the effects of multiply-fortified salt on the micronutrient status of school-aged children. The number and type of micronutrients included as fortificants in these studies have varied, but overall results have been promising. A trial of multiply-fortified salt containing vitamin A, B-1, B-2, B-6, niacin, iron, iodine, folic acid, B-12, and zinc conducted among students aged 5 to 18 y in 3 residential schools in southern India found that consumption of the fortified salt improved hemoglobin; ferritin; sTfR; body iron stores; and serum retinol, B-12, folate, and zinc compared with the control group (IS) after 9 mo supplementation [35]. Another RCT of multiply-fortified salt that contained chelated iron, iodine, vitamin B-12, and folic acid provided in noon meals to school children in India found improvements in biomarkers of iron status compared with a group that did not receive the fortified salt [36]. Although the present study did not observe differences in PSC iron or zinc status after 12 mo of consuming QFS, we did detect improvements in vitamin B-12 and folate status, as Vinodkumar et al. [35] and Kumar et al. [36] also reported.

Strengths of this study include that it was an RCT conducted in a community setting, so the results are more likely to represent a real-world setting. The 12 mo intervention period allowed us to assess micronutrient status at multiple time points. Unfortunately, it was not possible to measure discretionary salt intake directly, so we examined per capita household salt disappearance data instead, and we estimated salt intakes per child equivalent. Also, this study had a relatively small sample size and correspondingly limited power to assess the prevalence of micronutrient deficiencies using binary outcomes. At 33%, the actual attrition rate was higher than our anticipated attrition rate of 20%. Attrition was primarily due to blood draw refusal or failure; however, dropouts did not differ by intervention group. To address this, IPCW methods were used to allow researchers to assess study outcomes.

This was the first double-blinded, randomized, controlled, community-based trial to assess the effect of 2 types of QFS compared with IS on the micronutrient status of children aged 12 to 59 mo. Our study demonstrated modest improvements in markers of vitamin B-12 and folate status. This study suggests that QFS may be a useful strategy to address micronutrient deficiencies, especially vitamin B-12 and folate, among young children.

## Declaration of Generative AI and AI-Assisted Technologies in the Writing Process

The authors declare that no generative AI or AI-assisted technologies were used in the writing of this manuscript.

## Author contributions

The authors’ responsibilities were as follows – CMM, KHB, RD, MD, NFK: took responsibility for the design of the trial; YEG, MSM, BLS, GKB, LT: took responsibility for the implementation and oversight of data collection; MJ, JW: took responsibility for laboratory analyses; CDA: took responsibility for data analysis; JML: took primary responsibility for writing the manuscript, laboratory analyses, and data analysis; and all authors: read and approved the final version of the manuscript.

## Data availability

Data described in the manuscript, code book, and analytic code will be made available upon request, pending application, and approval.

## Funding

The study was funded by a grant from the Bill & Melinda Gates Foundation (INV 002945) and Thrasher Research Fund (grant 15463).

## Conflict of interest

The authors report no conflicts of interest.
