# Supplementary material for: Effects of Quintuply-Fortified Salt on the Micronutrient Status of Children 12 to 59 Months of Age in Punjab, India: Results from a Randomized, Community-Based, Household Trial
Source: J Nutr. 2026 May 5;156(7):101573. doi: 10.1016/j.tjnut.2026.101573 (PMC13494192; doi:10.1016/j.tjnut.2026.101573)
Supplement: Supplementary file 1 [file mmc1.docx]

**Supplementary Appendix**

**Supplementary Figure 1. Effect modification by baseline micronutrient status**
